# Influence of Preheating on the Microstructure of a Hot Extruded Nickel-Based Superalloy

**DOI:** 10.3390/ma18071478

**Published:** 2025-03-26

**Authors:** Jun-Cheng Zhu, Yong-Cheng Lin, Yun-Han Ling, Shu-Xin Li, Zi-Jian Chen, Yu-Liang Qiu

**Affiliations:** 1 School of Mechanical and Electrical Engineering, Central South University, Changsha 410083, China; 2 State Key Laboratory of Precision Manufacturing for Extreme Service Performance, Changsha 410083, China; 3 Rongcheng Huadong Metal-Forming Machinery Co., Ltd., Rongcheng 264300, China; 4 China Academy of Machinery Beijing Research Institute of Mechanical & Electrical Technology Co., Ltd., Beijing 100083, China; 5 School of Mechanical Engineering and Mechanics, Ningbo University, Ningbo 315211, China

**Keywords:** nickel-based superalloy, preheating, grain boundary, primary γ′ phase, dissolution kinetics

## Abstract

Some studies have reported the microstructure evolution of nickel-based superalloys during isothermal forging (IF). However, most of them have not taken into account the microstructure evolution during the preheating stage in manufacturing processes. Investigating the microstructure evolution mechanisms during preheating of nickel-based superalloy can provide a more accurate characterization of the initial microstructures prior to IF. In this study, the evolution of grain structure, participation phase, and twins in a hot extruded nickel-based superalloy are examined during heat treatment at the temperature range of 1050~1140 °C and 5~180 min. Also, the interaction mechanisms among the above microstructures are analyzed. Experimental results demonstrate that higher temperature significantly accelerates the dissolution of the primary γ′ (γ′_p_) phase and grain growth. At 180 min, the average grain size rapidly grows from 4.59 μm at 1080 °C to 14.09 μm at 1110 °C. In contrast, the impact of holding time on the microstructure diminishes after 30 min. At 1080 °C, the average grain size grows from 2.52 μm at 5 min to 4.95 μm at 30 min, after which it remains relatively stable. Initially, the γ′_p_ phase hinders grain boundary migration and inhibits grain growth. However, its complete dissolution at high temperatures significantly promotes grain growth. Careful selection of preheating temperature can mitigate rapid grain growth before forging. Additionally, twins not only refine grains through nucleation and segmentation, but also hinder grain boundary migration in regions with high dislocation density, thereby alleviating grain growth. A model detailing the dissolution of the γ′_p_ phase during preheating is developed, with a correlation coefficient and average absolute relative error of 0.9947 and 9.15%, respectively. This model provides theoretical support for optimizing preheating temperatures and estimating initial microstructures prior to IF.

## 1. Introduction

Nickel-based superalloys are well known for their exceptional mechanical performance, corrosion resistance, and fatigue properties, which make them the preferred material for manufacturing advanced aerospace engine turbine disks [1,2]. The outstanding performance of these alloys is largely attributed to their fine and uniform grain structure, along with the even distribution of strengthening particles [3,4,5,6]. Currently, large plastic deformations such as isothermal forging (IF) are the primary methods for processing these alloys [7,8]. Before hot deformation, billets must be preheated to the target temperature and maintained at this temperature for several hours to ensure uniform temperature distribution. However, during this preheating and holding phase, the microstructure of nickel-based alloys undergoes complex changes, resulting in an initial microstructure that differs from what is anticipated for the IF process. This discrepancy complicates accurate predictions of the microstructure and mechanical properties during IF. This research addresses this challenge through heat-treatment experiments designed to predict the microstructure during the preheating and holding stages of IF process. This research aims to optimize preheating parameters and provide superior initial microstructures for the IF process by studying the microstructure evolution after preheating.

In recent years, the hot deformation behavior of nickel-based superalloys has been extensively studied, with a primary focus on their flow behavior and microstructure evolution, leading to the development of corresponding predictive models [9,10,11,12]. Yang et al. [13] investigated the evolution of grain structure in a nickel-based superalloy at various temperatures and established a predictive model for grain size. Their findings revealed that the microstructure undergoes substantial changes near the solvus temperature, resulting in smaller grain sizes being preserved below this temperature. At temperatures above this threshold, grain growth becomes significant, which does not satisfy the size requirements for turbine disks. Therefore, this study limits the experimental temperature range to levels below the solvus temperature. Zhu et al. [14] developed a dynamic recrystallization (DRX) kinetic model for sub-solvus temperatures and found that the dissolution of the γ′_p_ phase significantly influences the DRX of nickel-based superalloys during hot deformation. Wen et al. [15] employed machine learning to establish a constitutive model for hot deformation, using a genetic algorithm-based artificial neural network (GA-ANN) to quantitatively describe the influence of deformation parameters and the volume fraction of the γ′_p_ phase on flow behavior. Chen et al. [16] formulated a unified constitutive model based on dislocation density. This model accounts for the effects of dislocation movement, grain boundaries, and precipitates, accurately predicting flow behavior at constant and variable strain rates. In summary, the initial microstructure, particularly the γ′_p_ phase, significantly influences the flow behavior and microstructure evolution of nickel-based superalloys [17,18]. However, many studies on hot deformation fail to consider the evolution of the γ′_p_ phase and other microstructures during preheating. Instead, these studies utilize the initial microstructure prior to preheating for modeling, resulting in inaccurate predictions from the models [19,20]. Therefore, investigating the evolution of microstructures during preheating can yield accurate initial microstructures for plastic deformation, thus establishing a reliable foundation for accurate predictions of flow behavior and microstructure evolution during deformation.

Several researchers have investigated the mechanisms and evolution of microstructures in nickel-based superalloys at high temperatures [21,22]. Wang et al. [23] investigated the evolution of the γ′ precipitate in nickel-based superalloys during heat treatment, finding that increasing temperature and prolonging holding time promote the dissolution of the γ′ precipitate. The γ′_p_ precipitate splits inward from the phase boundaries due to the elastic strain energy at these interfaces. Zhang et al. [24] observed that the dissolution of the γ′_p_ precipitate diminishes its “pinning effect” on grain boundaries during heat treatment, leading to accelerated grain growth. Li et al. [25] investigated the evolution of precipitates in a continuous cooling process after super-solvus heat treatment (SSHT) for FGH97 superalloy, and they found that the cooling rate greatly impacts the morphology and size of the secondary γ′ precipitates. Liu et al. [26] found that the initial microstructure greatly influenced the properties of K439B nickel-based superalloy aged at 800 °C for 1000 h. Cai et al. [27] developed a high-temperature pre-precipitation method to simultaneously form serrated grain boundaries and fine γ′ precipitates for a nickel-based powder superalloy, thus the mechanical properties are improved. Quan et al. [28] constructed a response surface for average grain size as a function of holding temperature and time using a BP-ANN model, describing grain growth behavior. Huang et al. [29] proposed a multiple-stage nucleation mechanism for γ′ phase during the continuous cooling of a nickel-based PM superalloy.

Many studies have reported that the γ′ phase dissolves during high-temperature holding [30,31], prompting inquiry into its sufficiency in preserving a relatively fine grain size after preheating and holding. Therefore, predicting the alloy’s microstructure post-preheating and holding through heat-treatment experiments can yield more accurate initial data for predictive models of microstructure during IF. Although numerous studies have examined the microstructure evolution of nickel-based superalloys during heat treatment, the majority focus on the heat treatment of the forged nickel-based alloys. Investigating the microstructure evolution mechanisms during preheating of nickel-based superalloy can provide a more accurate characterization of the initial microstructures prior to isothermal forging.

Therefore, in this work, some heat-treatment experiments are conducted on a hot extruded (HEXed) nickel-based superalloy. The effects of heat-treatment parameters on the grain structure and precipitation phases of the HEXed nickel-based superalloy are investigated. Additionally, the mechanisms of grain refinement and the interaction mechanisms among the microstructures are analyzed. A model detailing the dissolution of the γ′_p_ phase during preheating has been developed, offering theoretical support for optimizing preheating temperatures and estimating initial microstructures prior to IF.

## 2. Materials and Experiments

The chemical compositions of the studied material were determined by inductively coupled plasma–mass spectrometry (ICP-MS) technology, as shown in Table 1. The alloy is designated as FGH4113A alloy. The alloy powder was produced by initially melting the material under vacuum conditions, followed by argon atomization. To improve the quality of the powder, hollow particles and inclusions were removed, and powder with an average diameter of approximately 80 μm was selected. Subsequently, the powder was consolidated into billets through the hot isostatic pressing (HIP) process at 1150 °C under a pressure of 150 MPa for 4 h. After HIP treatment, the billets were extruded via a hot extrusion (HEX) process at 1110 °C, with an extrusion ratio of 4.7 and a speed of 35 mm/s, using a 5000-ton horizontal extrusion press.

Heat-treatment tests were conducted on the HEXed alloy in a muffle furnace (SX2-9-14TP, MITR Instrument & Equipment, Changsha, China) to investigate the evolution of grains and the γ′_p_ phases. The hearth of the muffle furnace is made of high-quality alumina ceramic fiber, ensuring high-temperature stability and excellent insulation performance, with a maximum operating temperature of up to 1400 °C. It is equipped with an advanced intelligent temperature control system, achieving a temperature control accuracy of ±1 °C. Cylindrical specimens with Φ 8 mm × 12 mm were extracted from an extruded billet of the nickel-based superalloy for the experiments. The furnace was preheated to the target temperatures. The samples were treated at 1050 °C, 1080 °C, 1110 °C, and 1140 °C for durations of 5 min, 30 min, 60 min, 120 min, and 180 min, respectively. After heat treatment, the specimens were quenched to maintain the deformed microstructure.

The heated samples underwent axial cuts for microstructure examination. Initially, the specimens were polished mechanically with diamond plaster to create a reflective surface suitable for scanning electron microscopy (SEM, TESCAN MIRA4, Brno, Czech Republic) observation. Subsequently, electrolytic polishing was performed with a solution of 30% H_2_O and 70% H_3_PO_4_ at room temperature for 30 to 50 s. This electrolyte selectively removes the γ matrix from the specimen surface while preserving the γ′ phase. To prepare for electron backscatter diffraction (EBSD, Oxford Instruments C-Nano, Oxford, United Kingdom) and transmission electron microscopy (TEM, Titan G2 60-300, Hillsboro, OR, USA) examinations, the specimen thickness was reduced to 60–100 μm through mechanical grinding. Standard 3 mm diameter discs were punched from the samples and underwent double-spray electrolysis using a mixture of 10% HClO_4_ and 90% ethyl alcohol. Electrolysis was conducted at −35 to −25 °C under a constant voltage of 20 V. For EBSD analysis, a scan region of 80 μm × 80 μm was employed with a step interval of 0.2 μm. HKL Channel 5 software 2019 was utilized for EBSD data analysis, while Velox software 3.13 was used for TEM observation.

## 3. Results

### 3.1. Microstructures of Extruded Alloy

**Figure 1:**
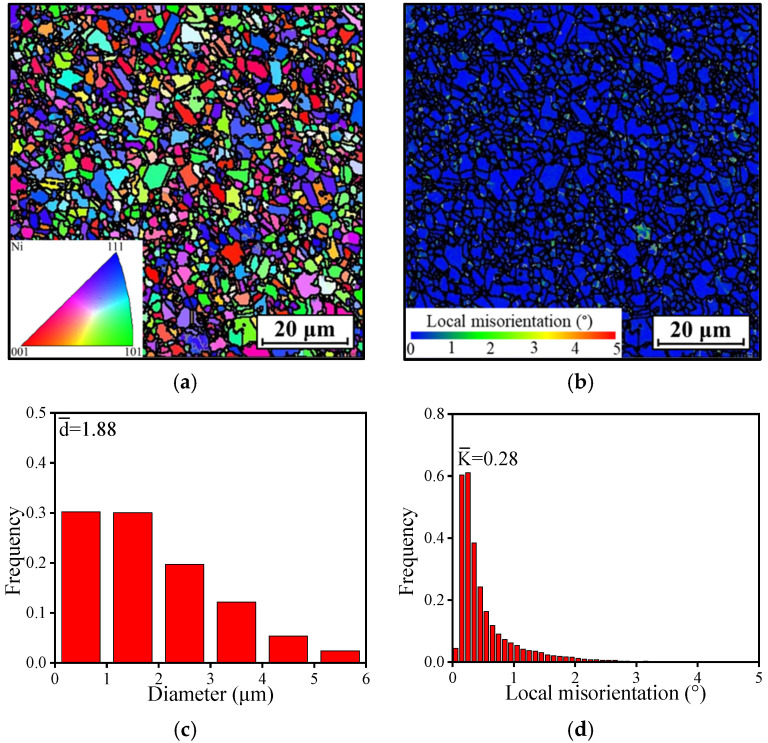
EBSD analysis of the superalloy: (**a**) IPF map; (**b**) KAM map; (**c**) grain size distribution; (**d**) local misorientation distribution.

Figure 1a illustrates the grain structure of the HEXed alloy as analyzed by EBSD. Here, the black lines signify grain boundaries, while the gray lines indicate sub-grain boundaries. After the HEX process, the alloy exhibits a reduced content of sub-grain boundaries, and the grains are notably fine. The grain size distribution was determined by volume fraction weighting, followed by the calculation of the average grain size. Figure 1c illustrates the detailed distribution of grain sizes that is shown in Figure 1a. It reveals that over 60% of the grains are less than 2 μm, with the maximum size not exceeding 6 μm and an average size of only 1.88 μm.

Figure 1b presents the kernel average misorientation (KAM) maps corresponding to Figure 1a, with colors representing the local misorientation at each position. Colors closer to green indicate a higher local misorientation within the grain, signifying an increased number of residual dislocations and higher localized strain energy, leading to decreased grain stability. Conversely, colors closer to blue indicate lower local misorientation. Figure 1d illustrates the specific distribution of local misorientation from Figure 1b, showing that most local misorientation values are below 1°, with an average local misorientation of only 0.28°. This low average local misorientation indicates that the alloy billet has undergone sufficient DRX during the HEX process, resulting in fewer residual dislocations within the alloy [32,33].

**Figure 2:**
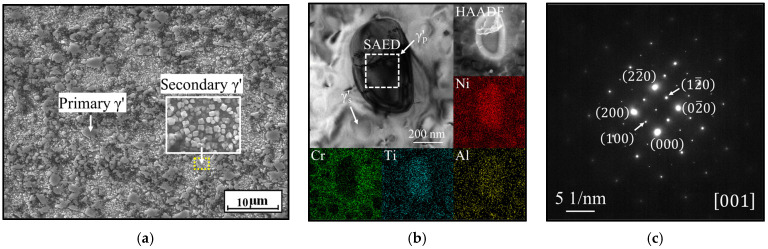
γ′ phase morphology in the superalloy: (**a**) SEM image; (**b**) element distribution (at. %); (**c**) SAED diagram.

Figure 2a illustrates the microstructure of the γ′ phases in the HEXed alloy, as observed through SEM. The alloy contains numerous γ′ phases, characterized by larger blocky γ′_p_ phases and smaller spherical γ′_s_ phases (the secondary γ′) that occupy the gaps between the γ′_p_ phases. The γ′_p_ phases are widely recognized to exert a pinning effect on grain boundaries during hot processing, which limits grain boundary migration and helps maintain fine grain structures [34]. Therefore, this study primarily focuses on the γ′_p_ phase, which accounts for approximately 40% of the volume fraction. The volume fraction of the γ′_p_ phase was quantified from SEM images magnified at 5000 times, analyzed using Image Pro Plus software 6.0. Figure 2b illustrates the elemental distribution around the γ′_p_ phase, and Table 2 presents the results of the EDS analysis for the main elements in γ′_p_ phase, revealing that the γ′ phase is primarily enriched in Al, Ti, and Ni, with a deficiency in Cr. The composition of the γ′ phase is Ni_3_ (Al, Ti), with Al, Ti, and Ni as the primary constituent elements, while Cr inhibits its precipitation behavior. Figure 2c is the selected-area electron diffraction (SAED) diagram from the boxed region in Figure 2b. The FCC structure and the extinction point reproduction due to its long-range ordered structure also indicate the characteristics of the γ′ phase [35,36,37]. This provides evidence for the identification of the γ′ phase in Section 4.1. The solvus temperature for the γ′ phase is 1150 °C. To investigate the impact of preheating below this temperature on the microstructure before forging, heat-treatment experiments in this study were conducted at temperatures below the solvus temperature.

### 3.2. Microstructure Evolution During Preheating

#### 3.2.1. Effect of Heating Temperature

**Figure 3:**
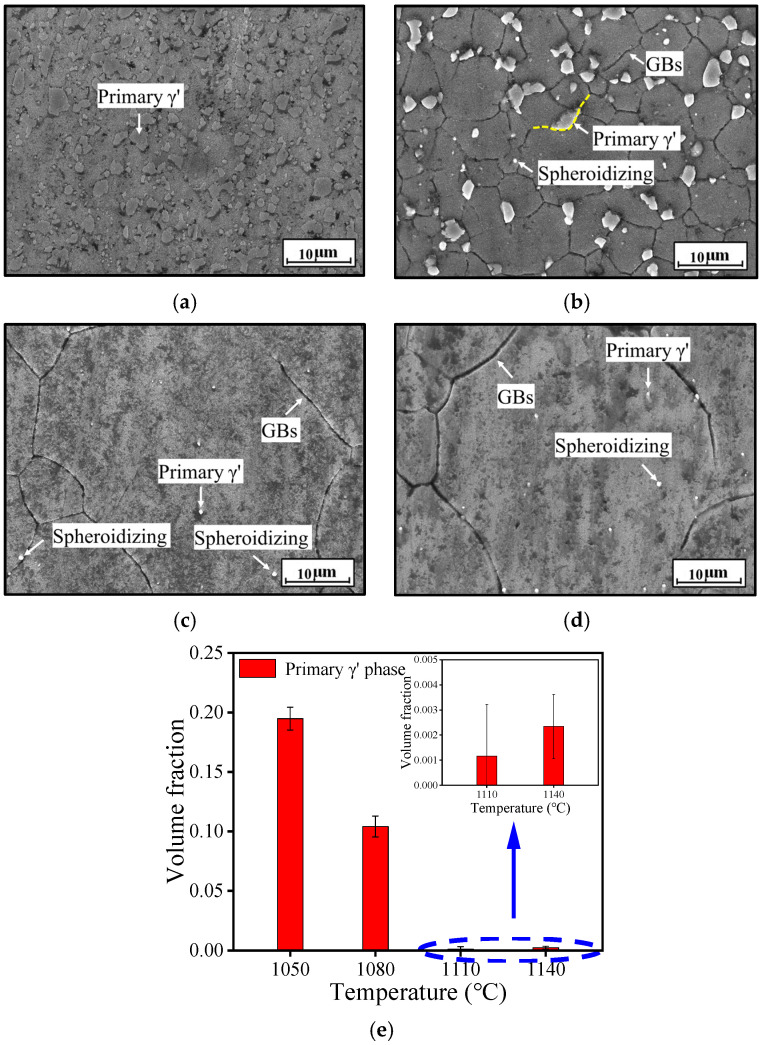
SEM images of the superalloy heated at (*t* = 180 min) (**a**) 1050 °C; (**b**) 1080 °C; (**c**) 1110 °C; (**d**) 1140 °C; (**e**) volume fraction of primary γ′ phase.

Figure 3 shows SEM images of the HEXed alloy heated at 1050 °C, 1080 °C, 1110 °C, and 1140 °C for 180 min; 180 min is selected as the standard because the preheating time of the billet in the manufacturing processes is generally about 180 min or longer. The γ′_s_ phase, commonly abundant in nickel-based superalloys, has completely dissolved and is no longer detectable. Figure 3e presents the volume fraction of the residual γ′_p_ phase after heat treatment at different temperatures. At 1050 °C, the alloy retains a substantial amount of γ′_p_ phase, which gradually decreases with increasing temperature, from 19.49% at 1050 °C to 10.41% at 1080 °C. At higher temperatures (1110 °C~1140 °C), only a minimal amount of γ′_p_ phase remains, with volume fractions falling below 0.5%. This indicates that the γ′_p_ phase has nearly entirely dissolved at these conditions. As the temperature increases, the reduction in the volume fraction of the γ′_p_ phase allows the grain boundaries (GBs) to be more easily etched. These SEM images also reveal considerable grain growth at higher temperatures. At lower temperatures (Figure 3b), the γ′_p_ phase predominantly appears in a blocky form along the grain boundaries. At higher temperatures (Figure 3c,d), the γ′_p_ phase transforms into a spheroidal morphology, distributed both at the grain boundaries and within the grains. This spheroidal shape results from spheroidization, a process that is facilitated by increasing temperature. However, the fine spheroidal γ′_p_ phases are less effective at pinning the grain boundaries, which permits some γ′_p_ phases to move into the grains as the boundaries migrate. Additionally, elevated temperatures promote element diffusion and enhance the driving force for grain boundary migration. An increase in temperature not only accelerates the dissolution of the γ′_p_ phases, but also enhances the interactions between grain boundaries and the γ′_p_ phases.

**Figure 4:**
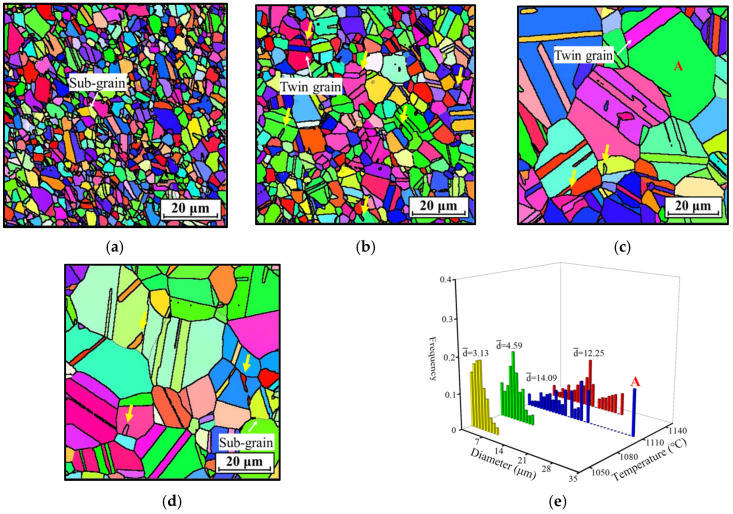
IPF maps of the HEXed nickel-based superalloy heated at (*t* = 180 min) (**a**) 1050 °C; (**b**) 1080 °C; (**c**) 1110 °C; (**d**) 1140 °C; (**e**) average grain size distribution. (The colors in (**a**–**d**) represent the grain orientations, the yellow arrows in (**b**–**d**) mark the fine twins, and the red letter “A” marks the unusually large grain).

**Figure 5:**
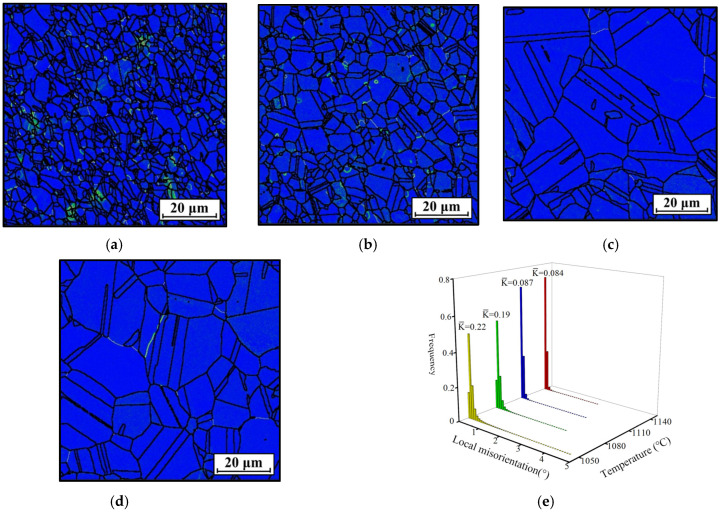
KAM figures of the HEXed nickel-based superalloy heated at (*t* = 180 min) (**a**) 1050 °C; (**b**) 1080 °C; (**c**) 1110 °C; (**d**) 1140 °C; (**e**) the statistical local misorientation. (The colors in (**a**–**d**) represent the local misorientation).

Figure 4 illustrates the inverse pole figure (IPF) maps of the nickel-based superalloy after being heated at different temperatures for 180 min. The grain structure exhibits straight grain boundaries with minimal distribution of fine grains. This suggests that DRX does not occur due to the absence of energy and dislocations generated by previous extrusion deformation during heat treatment, unlike in hot deformation. However, since the HEXed alloy retains some dislocations, static recrystallization (SRX) may occur [38]. Figure 4e presents the grain size distribution at various temperatures. The grain size gradually increases with rising temperatures due to enhanced grain boundary migration and the dissolution of the γ′_p_ phase, which progressively weakens its pinning effect on grain boundary movement [24]. Notably, at 1110 °C, the grain size significantly increases because the γ′_p_ phase has nearly completely dissolved (Figure 3c). This removes its pinning effect, allowing unrestricted grain growth. However, at 1140 °C, the grain size stabilizes and even slightly decreases, indicating that grain growth has reached a saturation point. Figure 5 displays the corresponding KAM maps at different temperatures. Figure 5e shows a statistical analysis of local misorientation. The average KAM values at all temperatures are lower than those of the HEXed alloy, primarily due to the absence of new dislocations generated during heat treatment. Instead, the residual dislocations from the initial microstructure are continuously consumed, facilitating grain growth. As the grain size increases, the average KAM value decreases with increasing temperature, showing a significant drop at 1110 °C, which is consistent with the grain size analysis. Above 1110 °C, the average KAM value falls below 0.1 and decreases only marginally with further increases in temperature. This indicates that the dislocations within the alloy have been almost entirely consumed, resulting in insufficient energy for additional grain growth and thus leading to saturation. At lower temperatures (1050 °C~1080 °C), the consumption of dislocations is incomplete, likely because the residual γ′_p_ phase restricts grain boundary migration and limits the alloy’s capacity to eliminate dislocations. In contrast, at higher temperatures (1110 °C~1140 °C), dislocations are almost fully consumed. Overall, although temperature affects grain size, grain growth is not directly controlled by heating temperature. Instead, variations in temperature influence the dissolution of the γ′_p_ phase, which in turn indirectly affects grain growth.

Figure 4 indicates that multiple twins are present in the alloy across all temperatures. These twins form during the grain growth process due to the misordered stacking of atomic layers [39]. The emergence of these fine twins facilitates grain refinement, as indicated by the yellow arrows. Moreover, the growth of twins leads to their traversal and division of the grains, which further decreases their size. In Figure 4c, a twin traversing grain A is positioned at the top, while another twin at the bottom has just nucleated and has not yet grown. This results in grain A being unusually large, consequently increasing the average grain size at 1110 °C (Figure 4e), even surpassing that at 1140 °C. Therefore, twinning is a crucial mechanism for grain refinement in the HEXed nickel-based superalloy.

The results indicate that after 180 min, the microstructure of the alloy exhibits significant differences compared to its initial state. This suggests that if the model mentioned in the Introduction is to be applied to the manufacturing processes of IF, it is essential to consider the effects of preheating.

#### 3.2.2. Effect of Holding Time

**Figure 6:**
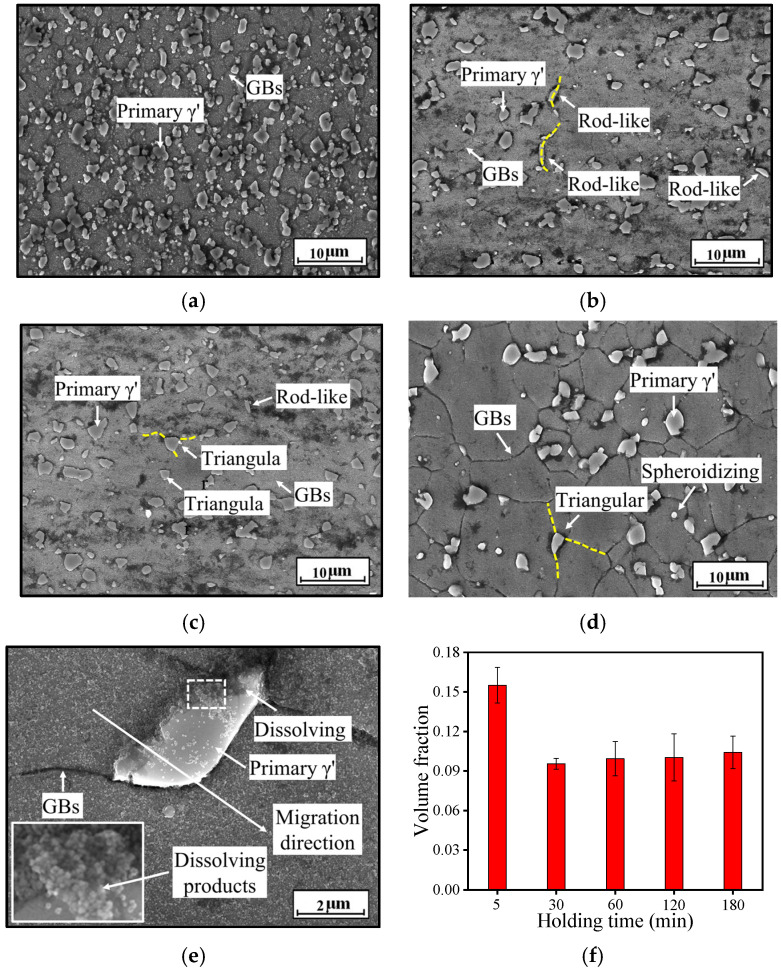
SEM micrographs of the HEXed nickel-based superalloy heated for (*T* = 1080 °C) (**a**) 5 min; (**b**) 30 min; (**c**) 60 min; (**d**) 120 min; (**e**) the enlarged view of Figure 3b (with the magnified image of the dotted white line in the lower-left corner); (**f**) volume fraction of primary γ′ phase.

Figure 6 presents SEM images of the superalloy heated at 1080 °C for 5 min, 30 min, 60 min, and 120 min, with the SEM image corresponding to 180 min displayed in Figure 6b; 1080 °C was selected as the standard because the alloy retains sufficient γ′_p_ phases and maintains fine grains at this temperature, as shown in Section 3.2.1. At 1080 °C, the alloy exhibits the γ′_p_ phases of uniform sizes across all holding time, while the γ′_s_ phases completely dissolve. Figure 6f illustrates the volume fraction of the residual γ′_p_ phases for different holding times. The results indicate that the volume fraction of the γ′_p_ phases rapidly decreases during the first 30 min, from 15.51% at 5 min to 9.55% at 30 min, and remains stable thereafter. Most γ′_p_ phases dissolve within the first 30 min of heat treatment, achieving saturation for γ′ phase-forming elements in the γ matrix. In Figure 6, some rod-like and triangular γ′_p_ phases are observed. The rod-like phases align along the grain boundaries (GBs), while the triangular phases are positioned at the vertices of the triangular grain boundaries, with the vertices directed toward the GBs (highlighted with yellow dashed lines). Figure 3b reveals a non-rod-like γ′_p_ phase dissolving at a grain boundary, accompanied by an enlarged image shown in Figure 6e. The grain boundary demonstrates a distinct tendency for downward migration, while the upper grain grows as a result of grain boundary movement. The γ′_p_ phase adjacent to the upper grain undergoes dissolution, resulting in numerous granular byproducts. Migration of the grain boundary disrupts the crystal structure, leading to the detachment of fine granular γ′ phases from the dissolving γ′_p_ phase. When the difference in dislocation density between the two grains decreases, the driving force for grain boundary migration diminishes. This limits grain growth, making the grain boundary straighter and resulting in narrow gaps. The dissolving γ′_p_ phase becomes confined between two grains, forming a flattened rod-like shape. The formation of triangular γ′_p_ phases, as shown in Figure 6, occurs through a comparable mechanism. These phases are located at the vertices of triangular grain boundaries, where multiple grains converge. The γ′_p_ phase experiences multi-directional forces, which result in its triangular shape. In the central region of the triangle, the more uniform multi-directional forces lead to slower dissolution, while the vertices experience stronger, unidirectional forces that promote faster dissolution and contribute to the triangular morphology. This suggests a reciprocal interaction: the γ′_p_ phase influences grain boundary migration, and conversely, grain boundary migration accelerates the dissolution of the γ′_p_ phase.

**Figure 7:**
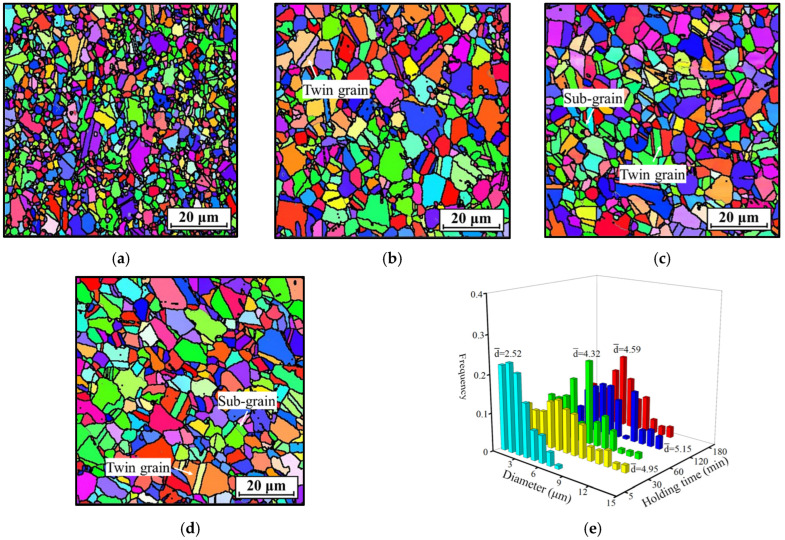
IPF maps of the HEXed nickel-based superalloy heated for (*T* = 1080 °C) (**a**) 5 min; (**b**) 30 min; (**c**) 60 min; (**d**) 120 min; (**e**) average grain size distribution. (The colors in (**a**–**d**) represent the grain orientations).

**Figure 8:**
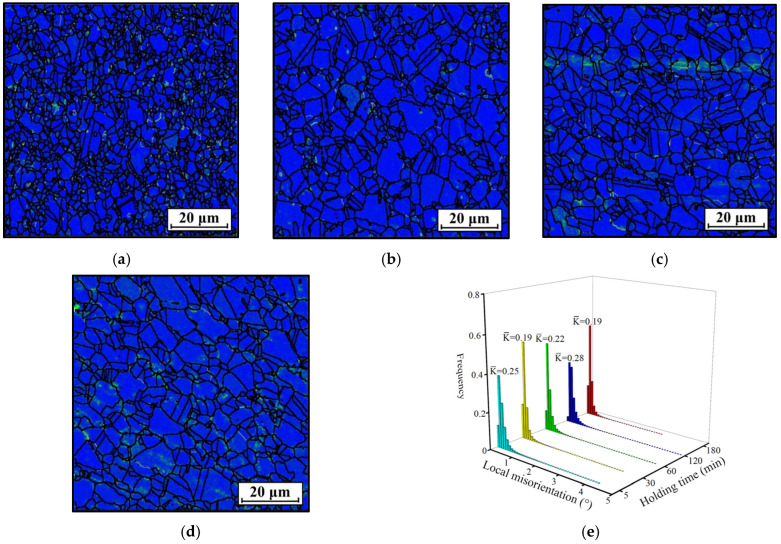
KAM figures of the HEXed nickel-based superalloy heated for (*T* = 1080 °C) (**a**) 5 min; (**b**) 30 min; (**c**) 60 min; (**d**) 120 min; (**e**) the statistical local misorientation. (The colors in (**a**–**d**) represent the local misorientation).

Figure 7 presents the IPF maps of the HEXed nickel-based superalloy subjected to holding times of 5 min, 30 min, 60 min, and 120 min at 1080 °C. The IPF map for 180 min at this temperature is presented in Figure 4b. Figure 7e depicts the grain size distribution at varying holding time. The grains grow rapidly during the first 30 min and then maintain a relatively constant grain size with extended holding time. Grain growth halts when the γ′_p_ phase ceases to dissolve. This indicates that the grain size is influenced by the volume fraction of the γ′_p_ phase. Moreover, the holding time is sufficient for grain growth and thus is not the limiting factor for it. This trend aligns closely with the alterations in the γ′_p_ phase; specifically, substantial dissolution of the γ′_p_ phase leads to rapid grain growth. Figure 8 shows the corresponding KAM maps for various holding time, while Figure 5b illustrates the KAM maps at 1080 °C/180 min. Figure 8e illustrates the statistical local misorientation. The average KAM value across different holding time significantly exceeds the minimum value observed in Figure 5e. This indicates that dislocations within the alloy remain partially present, and the absence of additional grain growth is not attributable to insufficient local strain energy, as described in Section 3.2.1. Instead, the γ′_p_ phase located at the grain boundaries, as depicted in Figure 6, exerts a pinning force that restricts the movement of migrating boundaries. Grain boundary migration is suppressed when the driving force is weaker than the pinning force exerted by the γ′_p_ phase. This prevents additional grain growth at a macroscopic level.

Previous studies suggest that when the sizes of the γ′_p_ phases are comparable, the magnitude of the pinning force is proportional to the volume fraction of the γ′_p_ phase [40]. Thus, the extent of permissible grain growth is influenced by the volume fraction of the γ′_p_ phase. Additionally, these studies indicate that within the experimental holding time range, the dissolution of the γ′_p_ phase tends to reach saturation and remains unchanged with increased holding time (Figure 6f). Therefore, after extensive grain growth during prolonged holding periods, the grain size is no longer affected by the holding time. Furthermore, grain boundary migration promotes the dissolution of the γ′_p_ phase, and this dissolution in turn promotes grain boundary migration. However, the cessation of both γ′_p_ phase dissolution and grain growth indicates that this mutual promotion does not occur simultaneously. This is because extending the holding time does not provide additional driving force for grain boundary migration to overcome the pinning force of the γ′_p_ phase. Additionally, the force exerted by the grain boundary during attempted migration is insufficient to promote γ′_p_ phase dissolution, and this effect does not accumulate with prolonged holding time. Figure 7 reveals numerous sub-grain boundaries, which are remnants of dislocations in the initial alloy that were rearranged during heating. At high temperatures, the movement of dislocations resumed, thereby facilitating SRX. While SRX contributes to grain refinement during heat treatment, the absence of relative grains rotations during hot deformation prevents the formation of large misorientation angles between sub-grain boundaries. As depicted in the corresponding KAM maps (Figure 8), dislocation accumulation and energy storage become more pronounced near these sub-grain boundaries. Sub-grain boundaries consume and dilute dislocations during their formation, thereby inducing dislocations to migrate away from grain boundaries. This reduces strain-induced boundary migration (SIBM) and mitigates grain growth. However, in the HEXed nickel-based superalloy, the sufficient DRX behavior results in reduced dislocation content (Figure 1b). Overall, SRX aids grain refinement during heat treatment by acting as a supplementary mechanism.

## 4. Discussion

### 4.1. Interaction Mechanism Between the γ′_p_ Phase and the Grain Structure

**Figure 9:**
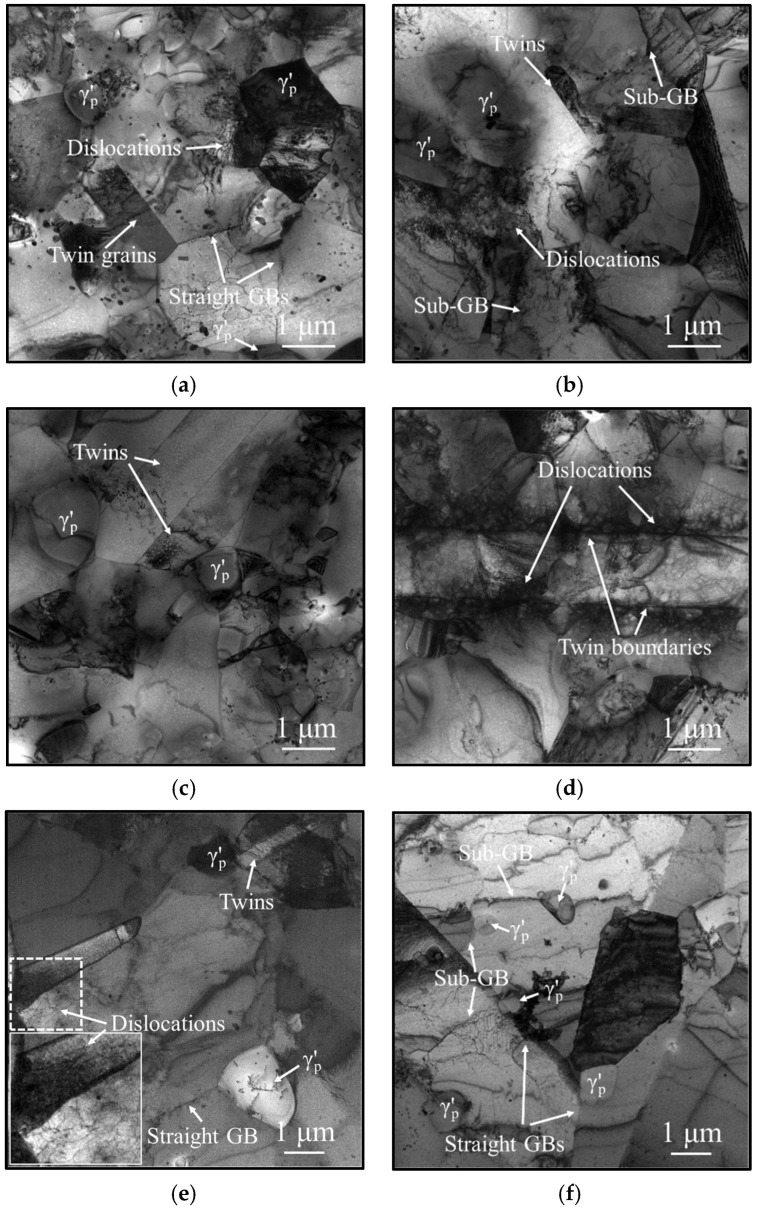
TEM micrographs of the HEXed nickel-based superalloy treated at (**a**–**d**) 1080 °C/30 min; (**e**,**f**) 1050 °C/180 min.

The grain refinement mechanism and its interaction mechanism with the γ′_p_ phase are further investigated using the TEM technique. Figure 9 shows TEM images of the HEXed nickel-based superalloy, subjected to varying temperatures and hold times. Typically, grain boundaries migrate toward the region characterized by higher dislocation density and strain energy storage, resulting in the consumption of dislocations and a reduction in strain energy. However, as depicted in Figure 9a,e,f, numerous γ′_p_ phases are distributed along the grain boundaries. These boundaries remain linear and do not bow toward the region of higher dislocation density. This occurs because when the grain boundary moves away from the γ′_p_ phase, the interfacial area increases, resulting in higher interfacial energy. To prevent this energy increase, the γ′_p_ phase exerts a traction force on the grain boundary, impeding its migration and consequent grain growth. This phenomenon is known as the “pinning effect”.

In Figure 9a,b, numerous dislocations accumulate around the γ′_p_ phases due to the complex structure at the phase boundaries, which impedes dislocation movement. A high dislocation density induces dislocation rearrangement and SRX, which reduces local strain energy. Consequently, sub-grain boundaries form around the γ′_p_ phases, as observed in Figure 9b,f. In Figure 9a–c, extensive twinning occurs around the γ′_p_ phases where complex stress facilitates the misordering of atomic layers during grain growth. In Figure 9d, numerous dislocations accumulate along the twin boundaries without being consumed. Twin boundaries not only impede the migration of adjacent grain boundaries, but also remain stationary. Therefore, the twins exhibit high stability during heating. Twinning in regions of high dislocation density effectively mitigates grain growth induced by SIBM [41]. In Figure 9e, dislocations are enclosed by growing twins, rendering them ineffective for SIBM. Additionally, the growth of twins consumes dislocations present in the original grains and simultaneously promotes grain refinement. In summary, the γ′_p_ phase not only directly restricts grain growth through the pinning effect, but also enhances grain refinement by promoting the nucleation of sub-grains and twins.

**Figure 10:**
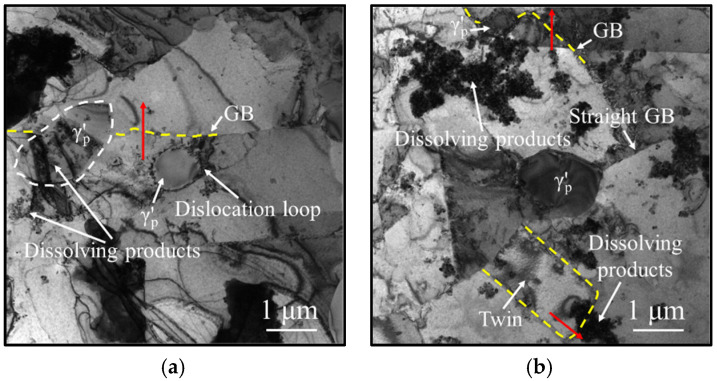
TEM micrographs of the HEXed nickel-based superalloy heated at 1050 °C for 180 min. The interaction between the γ′p phases and: (**a**) grain boundaries; (**b**) twins. (The dotted lines mark the grain boundaries, and the red arrows mark the migration directions of the grain boundaries).

Figure 10 illustrates the effect of grain boundaries on the γ′_p_ phases. The grain boundaries, indicated by yellow dashed lines, traverse the γ′_p_ phases and exhibit upward curvature. Multiple spheroidal dissolution products are dispersed around the γ′_p_ phases. Grain growth and grain boundary migration promote the dissolution of the γ′_p_ phases. In Figure 10a, the grain boundary completely bypasses a smaller γ′_p_ phase, leaving a dislocation loop along its migration path. This observation confirms that the interaction between grain boundaries and the γ′_p_ phases follows the Orowan mechanism. Furthermore, in Figure 10b, twin growth further promotes the dissolution of the γ′_p_ phases.

### 4.2. γ′_p_ Phase Dissolution Kinetics Models

As the preheating temperature increases from 1080 °C to 1110 °C, the grain size within the alloy increases significantly more than during the temperature range of 1050 °C to 1080 °C, due to the complete dissolution of the γ′_p_ phase (Figure 4). The results of this evaluation demonstrate that the γ′_p_ phase significantly influences the evolution of grain structure. Sufficient γ′_p_ phases maintain the grain structure’s pinning effect, preventing excessive growth during preheating. The dissolution behavior of the γ′_p_ phase directly influences whether the grain structure of the HEXed nickel-based superalloy remains fine and uniform prior to forging post-preheating. Previous studies reveal that, at a constant heat-treatment temperature, the volume fraction of the γ′_p_ phase decreases rapidly in the initial stages before stabilizing. This observation conforms to the following Johnson–Mehl–Avrami–Kolmogorov (JMAK) model [23,30],

(1) $$f_{{\gamma^{\prime }}_{p}}=f_{\mathrm{eq}}+f_{1}\exp\left(-\frac{t}{t_{1}}\right)$$

where $f_{{\gamma^{\prime }}_{p}}$ represents the volume fraction of the γ′_p_ phase at the target heat-treatment parameters, $f_{\mathrm{eq}}$ denotes the volume fraction of the γ′_p_ phase at thermodynamic equilibrium, t is the holding time (min), and $f_{1}$ and $t_{1}$ are material parameters. It is evident that a single JMAK model can only predict $f_{{\gamma^{\prime }}_{p}}$ for different holding time at a specific constant temperature. To predict $f_{{\gamma^{\prime }}_{p}}$ at different heating temperatures, distinct models are necessary for each temperature. At 1110 °C, the γ′_p_ phase completely dissolves in the stable stage, indicating that the dissolution kinetics models for γ′_p_ phase focus solely on changes occurring at 1050 °C, 1080 °C, and 1110 °C.

**Figure 11:**
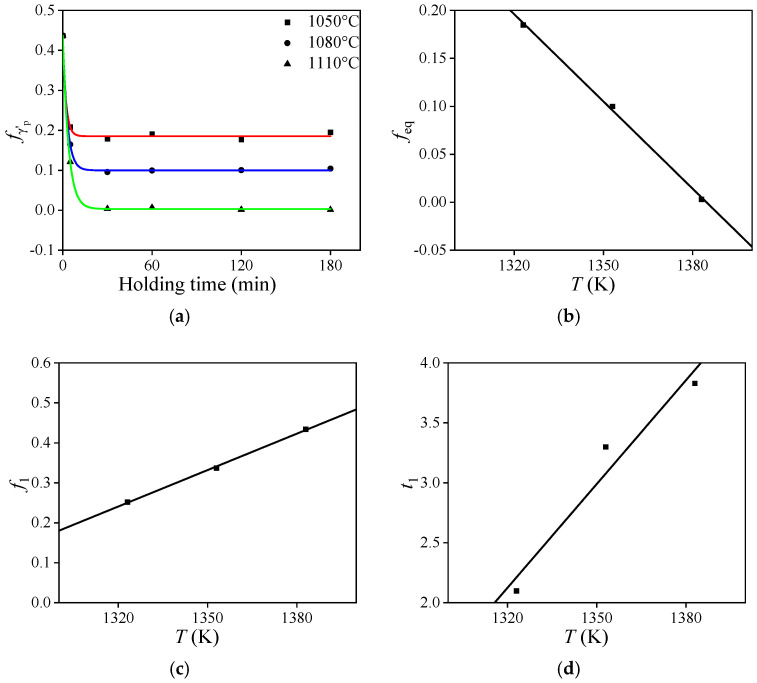
The relationship between (**a**) $f_{{\gamma^{\prime }}_{p}}$− t; (**b**) $f_{\mathrm{eq}}$ − T; (**c**) $f_{1}$ − T; (**d**) $t_{1}$ − T.

Figure 11a presents the variation in $f_{{\gamma^{\prime }}_{p}}$ with holding time during heat treatment at 1050 °C, 1080 °C, and 1110 °C, along with the corresponding fitted curves generated using Origin 2021 software. The fitting results are as follows:

(2) $$f_{{\gamma^{\prime }}_{p}}=\left\{\begin{array}{l} 0.185+0.252\exp\left(-\frac{t}{2.10}\right)\;\mathrm{for}\;1050\;{}^{\circ}C\\ 0.100+0.337\exp\left(-\frac{t}{3.30}\right)\;\mathrm{for}\;1080\;{}^{\circ}C\\ 0.003+0.434\exp\left(-\frac{t}{3.83}\right)\;\mathrm{for}\;1110\;{}^{\circ}C \end{array}\right.$$

As illustrated in Figure 11b–d, a good linear relationship exists between $f_{\mathrm{eq}}$, $f_{1}$ and $t_{1}$ with the heating temperature (*T*) across various temperatures. $f_{\mathrm{eq}}$ decreases linearly with increasing temperature, indicating a linear relationship between the equilibrium $f_{{\gamma^{\prime }}_{p}}$ and temperature before the γ′_p_ phase completely dissolves, assuming adequate preheating time. These findings serve as a reference for preheating studies on the nickel-based superalloy prior to various hot forming processes. The preheating results can be obtained through direct linear fitting, thereby eliminating the need for multiple heat-treatment experiments with varying preheating time and JMAK model adjustments. Linear fitting allows for the expression of the γ′_p_ phase dissolution kinetics models under various heating temperatures and holding times, as follows:

(3) $$\left\{\begin{array}{l} f_{{\gamma^{\prime }}_{p}}=f_{\mathrm{eq}}+f_{1}\exp\left(-t/t_{1}\right)\\ f_{\mathrm{eq}}=-0.00303T+4.200\\ f_{1}=0.00303T-3.763\\ t_{1}=0.02883T-35.935 \end{array}\right.$$

**Figure 12:**
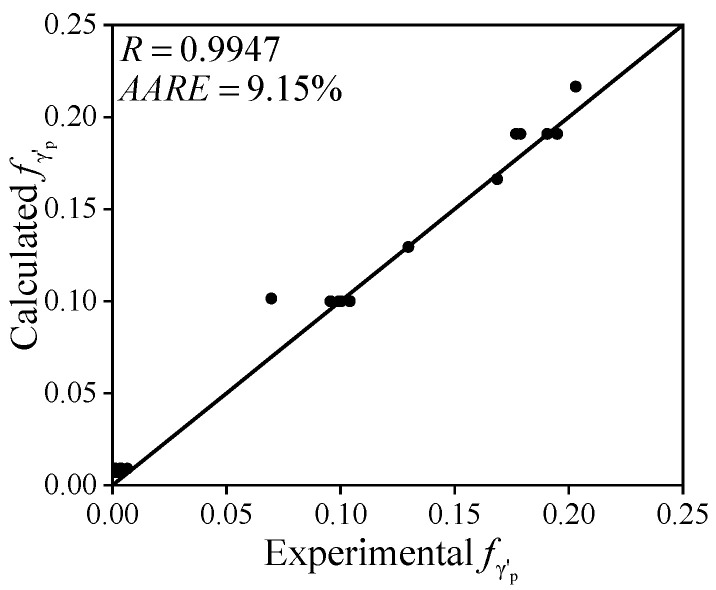
The comparisons between the calculated and experimental $f_{{\gamma^{\prime }}_{p}}$ (marked by the dots).

Figure 12 presents a comparison between the calculated and observed values of $f_{{\gamma^{\prime }}_{p}}$. The correlation coefficient (*R*) is 0.9947, with an average absolute relative error (*AARE*) of 9.15%. The high *R* values and small errors demonstrate that the model accurately predicts the volume fraction of γ′_p_ phase during preheating across various temperatures before forging.

## 5. Conclusions

The dissolution behavior of the γ′_p_ phase and the evolution of grain structure in the HEXed nickel-based superalloy during preheating prior to forging are investigated. The impact of the γ′_p_ phase on grain structure is discussed. The findings lead to the following significant conclusions:

(1) Increasing the temperature markedly accelerates grain growth and dissolution of the γ′_p_ phases. At 180 min, the volume fraction of the γ′_p_ phase decreases from 19.49% at 1050 °C to 10.41% at 1080 °C, while the grain size grows from 3.13 μm to 4.59 μm. However, beyond 1110 °C, both phenomena reach saturation. Extended holding time exerts a limited effect, primarily within the first 30 min. At 1080 °C, the volume fraction of the γ′_p_ phase decreases from 15.51% at 5 min to 9.55% at 30 min, while the grain size grows from 2.52 μm to 4.95 μm. Temperature and time indirectly influence grain growth by regulating the γ′_p_ phases.

(2) During preheating, twinning is the primary mechanism for grain refinement. However, improper twin growth positions can significantly increase the average grain size, resulting in a larger grain size of 14.09 μm at 1110 °C, compared to 12.25 μm at 1140 °C. Residual dislocations in the initial alloy undergo SRX, but due to the low dislocation density, this process serves only as an auxiliary means for grain refinement.

(3) The undissolved γ′_p_ phase significantly inhibits grain growth while promotes twinning and SRX, thereby further refining the grains. Grain boundary migration, caused by grain growth and twin growth, also promotes the dissolution of the γ′_p_ phase.

(4) The γ′_p_ phase has a significant impact the microstructure of the nickel-based superalloy. The dissolution model of the γ′_p_ phase provides a foundation for predicting its initial content during the forging process. The JMAK model developed in this study accurately predicts variations in the volume fraction of the γ′_p_ phase during the preheating process, as evidenced by the high *R* of 0.9947 and the low AARE of 9.15%. Modeling results indicate that the equilibrium volume fraction of the γ′_p_ phase exhibits a good linear relationship with temperature when preheating time is sufficient.

These conclusions demonstrate that preheating has a significant impact on the microstructures of nickel-based superalloys prior to IF. The complex interaction mechanisms between microstructures cause them to continuously evolve during the preheating process. This research provides a better understanding and prediction of the microstructures of nickel-based superalloys after preheating, ensuring the accuracy of microstructure prediction models during IF. Although this study discusses the pinning effect of the γ′_p_ phase and observes its failure at 1110 °C, further research is still required to quantitatively link the failure conditions to the volume fraction of the γ′_p_ phase.

## Figures and Tables

**Table 1:** Chemical composition of the studied material (wt. %).

Co	Cr	Mo	W	Ti	Al	Nb	Ta	Hf	Ni
19.1	12.9	4.1	4.1	3.7	3.0	1.3	1.0	0.2	Bal.

**Table 2:** EDS analysis results for main elements in the γ′_p_ phase (at. %).

Ni	Co	Ti	Al	Cr	Mo	Nb	Ta	W	Hf
62.82	12.68	8.81	7.76	3.76	1.22	1.05	0.99	0.79	0.11

## Data Availability

The raw data supporting the conclusions of this article will be made available by the authors on request.
